# Supplementary material for: The causal role of circulating inflammatory markers in osteoporosis: a bidirectional Mendelian randomized study
Source: Front Immunol. 2024 Jul 18;15:1412298. doi: 10.3389/fimmu.2024.1412298 (PMC11291241; doi:10.3389/fimmu.2024.1412298)
Supplement: Supplementary file 1 [file DataSheet_1.zip › supplementary material/CIM-OSTEOPOROSIS /GCST90274827.csv_forest.pdf]

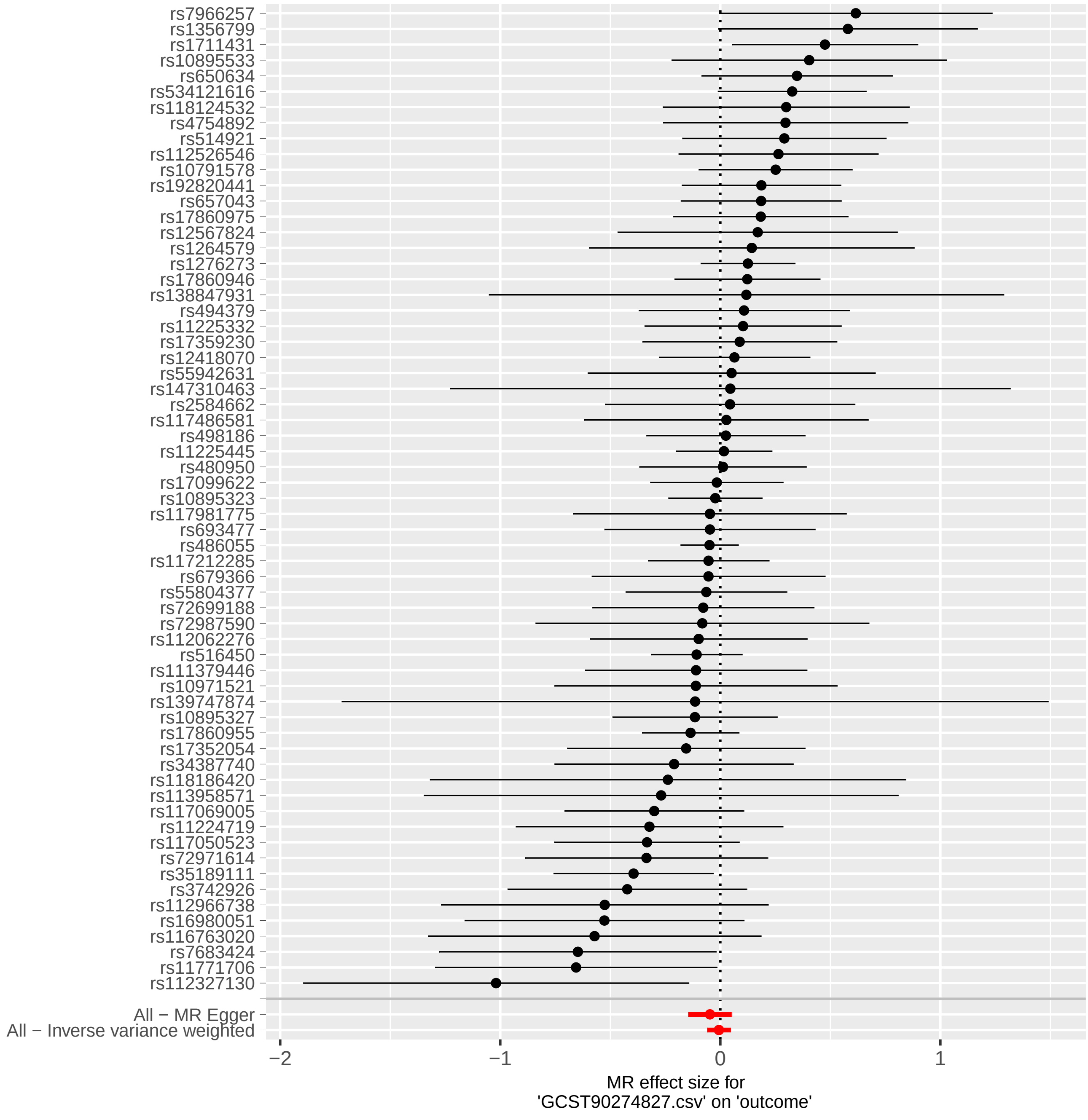
rs7966257
rs1356799
rs1711431
rs10895533
rs650634
rs534121616
rs118124532
rs4754892
rs514921
rs112526546
rs10791578
rs192820441
rs657043
rs17860975
rs12567824
rs1264579
rs1276273
rs17860946
rs138847931
rs494379
rs11225332
rs17359230
rs12418070
rs55942631
rs147310463
rs2584662
rs117486581
rs498186
rs11225445
rs480950
rs17099622
rs10895323
rs117981775
rs693477
rs486055
rs117212285
rs679366
rs55804377
rs72699188
rs72987590
rs112062276
rs516450
rs111379446
rs10971521
rs139747874
rs10895327
rs17860955
rs17352054
rs34387740
rs118186420
rs113958571
rs117069005
rs11224719
rs117050523
rs72971614
rs35189111
rs3742926
rs112966738
rs16980051
rs116763020
rs7683424
rs11771706
rs112327130
All − MR Egger
All − Inverse variance weighted
−2
−1
0
1
MR effect size for
'GCST90274827.csv' on 'outcome'
